# Peripartum Isolated Cortical Vein Thrombosis in a Mother with Postdural Puncture Headache Treated with an Epidural Blood Patch

**DOI:** 10.1155/2013/701264

**Published:** 2013-03-03

**Authors:** Etienne Laverse, Sarah Cader, Rajith de Silva, Sanjiv Chawda, Satish Kapoor, Olaleye Thompson

**Affiliations:** ^1^Department of Neurology, Queen's Hospital, Romford RM7 0AG, UK; ^2^Department of Neuroradiology, Queen's Hospital, Romford RM7 0AG, UK; ^3^Department of Anaesthetics, Queen's Hospital, Romford RM7 0AG, UK; ^4^Department of Obstetrics & Gynaecology, Queen's Hospital, Romford RM7 0AG, UK

## Abstract

A 32-year-old woman presented with low pressure headache 3 days after delivery of her baby. An assessment of postdural puncture headache was made. This was initially treated with analgesia, caffeine, and fluids for the presumed cerebrospinal fluid (CSF) leak. The woman was readmitted two days after her hospital discharge with generalised seizures. A brain scan showed features of intracranial hypotension, and she was treated for CSF leak using an epidural blood patch. Her symptoms worsened and three days later, she developed a left homonymous quadrantanopia. An MRI scan confirmed a right parietal haematoma with evidence of isolated cortical vein thrombosis (ICVT).

## 1. Introduction

Thrombosis is the third leading cause of death during pregnancy and the puerperium. Although rare, cerebral venous thrombosis (CVT) is a significant cause of maternal mortality. CVT remains a diagnostic challenge, due mainly to the wide variation in its clinical presentation. Headache being a key presenting feature, the differential diagnoses can be extensive, making accurate diagnosis difficult. It typically occurs in young otherwise previously healthy women and is therefore highly significant because it can result in serious neurological sequelae or death. Such deaths are potentially preventable with heightened vigilance and earlier diagnosis and treatment.

We report a case of postpartum isolated cortical vein thrombosis (ICVT) in the context of post-dural puncture headache treated with an epidural blood patch. A heightened awareness of this condition following the recent Centre for Maternal and Child Enquiries (CMACE) morbidity and mortality reports and the utilisation of appropriate imaging techniques enabled a timely diagnosis. The risk factors for CVT and the potential for missed or delayed diagnosis in the context of post-dural puncture headache are discussed.

## 2. Case Presentation

A previously healthy 32-year-old woman developed severe frontal headache following a spontaneous labour. Epidural analgesia had been administered during labour. She was readmitted three days later with persistent postural headache, which was treated with analgesia, caffeine, and fluids, and she was discharged home with improvement in symptoms. 

She was readmitted again, two days following the second hospital discharge, having had two seizures at home (an initial focal seizure, followed ten minutes later by a second, generalised tonic-clonic convulsion). Her headache which remained postural, being worse on sitting or standing, continued and she was treated for a persistent CSF leak with a blood patch. A sudden deterioration followed this with the development of a left homonymous quadrantanopia three days later. 

**Figure 1:**
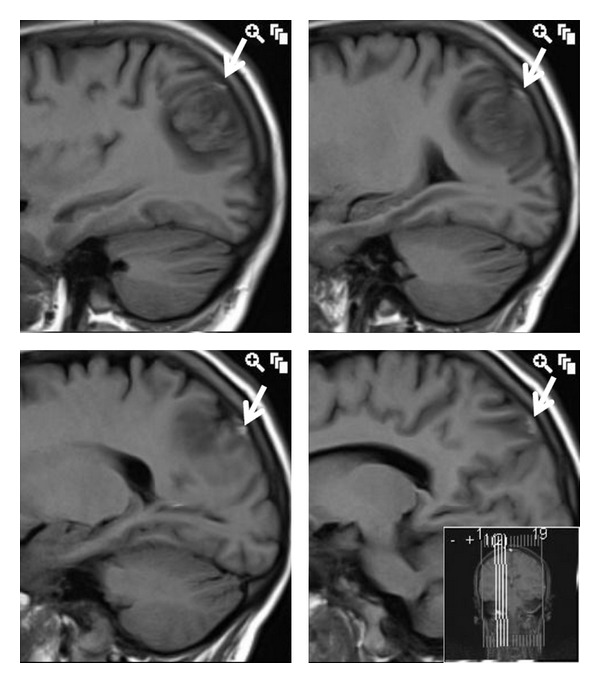
T1W sagittal image showing tubular hyperintense thrombus of the draining superior cortical vein (white arrows) posterosuperior to the haematoma.

**Figure 2:**
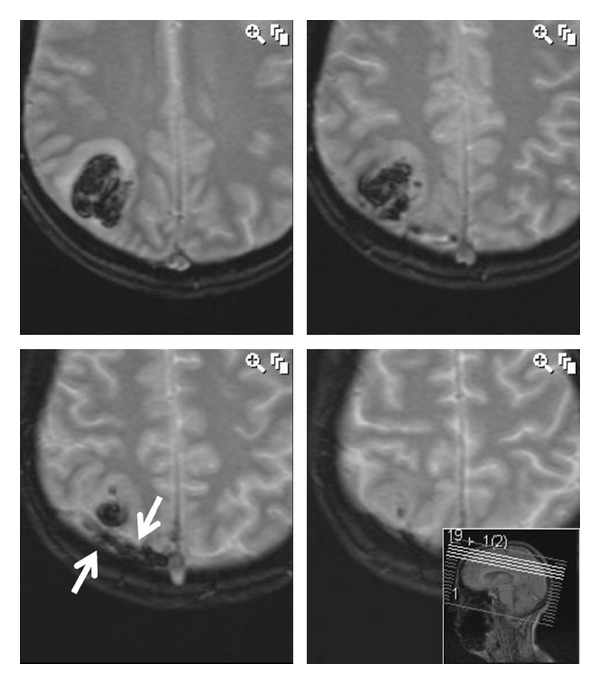
Gradient Echo T2W image showing a 3 cm haematoma and surrounding oedema in the right parietal lobe. White arrowindicating the hypointense thrombosed vein.

The initial CT showed a 5 mm focus of haemorrhage in the right parietal lobe but no “cord sign” to suggest cortical vein thrombosis. MRI following the blood patch showed a 3 cm area of haemorrhage and surrounding oedema in the right parietal lobe with a high T1 signal vessel in relation to the haematoma that represents the thrombosed vein (Figure 1). The corresponding Gradient Echo T2-weighted images (Figure 2) show a low T2 signal intensity structure adjacent to the haematoma also representing the thrombosed vein. The MRV and CTV showed the major dural sinuses to be patent including the superior sagittal sinus with the thrombosed cortical vein not seen to fill with contrast.

Antiepileptic medication (Levetiracetam) was prescribed for seizure control, and the thrombosis was managed with a six-month course of Warfarin therapy. The symptoms resolved completely and the woman has remained well. There have been no further seizures and the left field quadrantanopia resolved with no neurological sequelae. 

## 3. Discussion

In the most recent triennial confidential enquiry into maternal deaths in the UK, thromboembolic disorders, as a group, constitute the 3rd leading cause of direct maternal deaths at a rate of 0.79 women per 100,000 maternities [1]. The reported incidence of cerebral venous thrombosis in pregnancy is in the range of 1 : 10000 to 1 : 25000, and there are around 7 reported cases in postpartum patients following dural puncture [2, 3]. A case of isolated CVT (i.e., without sinus involvement) appears extremely rare and has been mainly reported as case reports or in small series [4].

Primary headaches, for example, migraine, can improve during pregnancy while symptomatic or secondary headaches, for example, due to cerebral venous thrombosis, may increase. Previous confidential enquiries into maternal deaths have therefore recommended that severe, persisting headache must be considered an indication for urgent neuroimaging [1] so that thromboses could be diagnosed as an emergency and anticoagulation could be given urgently. 

MRI is the most appropriate diagnostic tool as it may enable direct visualization of the thrombus in the affected superficial cortical vein through marked hypointensity on T2- weighted-gradient-echo image (T2*), hyperintensity on T1 weighted image, as well as the secondary changes of venous outflow obstruction seen in the form of swollen gyri as a result of venous congestion. The use of a T2*-weighted gradient-echo (T2*GE) sequence is very sensitive to all paramagnetic products of haemoglobin and is particularly useful for early diagnosis of CVT [4]. The CT may show a “cord sign” (i.e., cortical hyperdensity in relation with thrombosis of a cortical vein) [4].

Epidural analgesia is widely used in labour and post-dural puncture headache (PDPH), following epidural insertion, is a known complication [2]. Headache is also a common symptom of CVT [5, 6] and can therefore make early diagnosis, in the context of peripartum dural puncture, difficult. 

A sudden decrease in CSF pressure after dural puncture is thought to activate brain adenosine receptors leading to vascular dilatation and therefore PDPH [2]. Low CSF pressure causes downward traction on the cortical sinuses and veins, which may lead to thrombosis [6]. An epidural blood patch occludes the dural puncture and stops further CSF leakage. In this case, an ICVT was diagnosed after an epidural blood patch was performed, which was reflected in the deterioration in her symptoms following the procedure.

Precipitating factors for thrombosis include infection, trauma, pregnancy, and the puerperium [5]. There may also be predisposing genetic and acquired prothrombotic factors [5, 6]. As PDPH and CVT occur early in the postpartum period, late diagnosis and inadequate treatment can readily result [7]. The patient's initial headache had a marked postural component and developed within 48 hours of a dural puncture, making an accurate diagnosis of PDPH highly likely [8]. However, the seizures that occurred before the blood patch procedure may have been caused by CSF leakage, caffeine [9], or indeed an undiagnosed ICVT. 

The presenting symptoms of CVT can be grouped into four major syndromes, isolated intracranial hypertension, focal syndrome, encephalopathy, and cavernous sinus syndrome [5]. Although confirmatory diagnostic imaging was performed after the blood patch procedure, it remains possible that the seizures in this case, after conservative management of a CSF leak, were part of the focal syndrome. Yet with the sudden deterioration in her symptoms and the development of the quadrantanopia soon after the blood patch procedure, it is likely that the ICVT was made worse by the blood patch against a background setting of iatrogenic intracranial hypotension [3].

The management of CVT includes anticoagulation with Heparin to stop the thrombotic process [6]. Most patients are treated with Warfarin for six months thereafter. Case reports have reported successful use of endovascular thrombolysis in patients who deteriorate following standard anticoagulation [6].

A diagnosis of CVT or ICVT in the context of peri-partum postlumbar puncture syndrome and an epidural blood patch can be difficult to establish. CVT should always be suspected particularly if symptoms, such as headache, fail to improve or, as in our case, deteriorate after a blood patch procedure. Diagnostic MRI and MRV imaging should be carried out as soon as possible as the management is usually straightforward and can avert serious neurological sequelae.

*Key Points*

- PDPH, a low pressure/volume headache, is a known complication of epidural analgesia.
- PDPH can be managed with an epidural blood patch.
- CVT has several precipitants in the obstetric population and should be excluded early, when headaches or other neurological signs and symptoms are present.
- MRI is the most appropriate diagnostic tool for ICVT as it may enable direct visualization of the thrombus in the affected superficial cortical vein and anticoagulation is the mainstay of treatment.

## References

[1] Cantwell R, Clutton-Brock T, Cooper G (2011). Saving Mothers’ Lives: reviewing maternal deaths to make motherhood safer: 2006–2008. The Eighth Report of the Confidential Enquiries into Maternal Deaths in the United Kingdom. *British Journal of Obstetrics and Gynaecology*.

[2] Ghatge S, Uppugonduri S, Kamarzaman Z (2008). Cerebral venous sinus thrombosis following accidental dural puncture and epidural blood patch. *International Journal of Obstetric Anesthesia*.

[3] Kinney MO, McCarron MO (2011). Intracranial hypotension and venous sinus thrombosis: two postpartum headaches. *Postgraduate Medical Journal*.

[4] Boukobza M, Crassard I, Bousser MG, Chabriat H (2009). MR imaging features of isolated cortical vein thrombosis: diagnosis and follow-up. *American Journal of Neuroradiology*.

[5] Ferro JM, Canhão P (2003). Cerebral venous and dural sinus thrombosis. *Practical Neurology*.

[6] Anand N, Chan C, Wang NE (2009). Cerebral venous thrombosis: a case report. *Journal of Emergency Medicine*.

[7] Borum SE, Naul LG, McLeskey CH (1997). Postpartum dural venous sinus thrombosis after postdural puncture headache and epidural blood patch. *Anesthesiology*.

[8] Goadsby PJ, Boes C, Sudlow CLM (2002). Low CSF volume (pressure) headache. *Practical Neurology*.

[9] van Nordennen J, Camu F (2007). Postpartum seizures after epidural analgesia: a patient with a mutation of the factor V Leiden and prothrombin gene. *Journal of Clinical Anesthesia*.

